# Bacterial diadenylate cyclase domains synthesize diverse nucleotide signals in anti-phage defense

**DOI:** 10.64898/2026.08.10.743927

**Published:** 2026-08-11

**Authors:** Adelyn E. Ragucci, Danielle J. Haley, Philip J. Kranzusch

## Abstract

Bacterial diadenylate cyclase (DAC) enzymes synthesize the nucleotide signal 3′3′ cyclic di-AMP (3′3′-c-di-AMP) to control osmoregulation, cell-wall homeostasis, and DNA-damage responses. Here we discover specialized roles for DAC enzymes in bacterial immunity and define a Panoptes-like system we name Panoptoo as a DAC-containing anti-phage defense that guards against viral immune evasion. The Panoptoo protein PanS is a minimal DAC that constitutively synthesizes 3′3′ cyclic UMP-AMP (3′3′-cUA) or 3′3′-c-di-AMP to negatively regulate a partnering PanE S2TMβ membrane-targeting effector. We show that Panoptoo decoy signaling acts as a counter-defense to detect phage immune evasion proteins that inhibit nucleotide immune signals. A 1.5 Å crystal structure of PanS in complex with 3′3′-cUA explains how a symmetry break in the canonical DAC active site enables synthesis of asymmetric signaling molecules. Together, our results uncover a role for DAC domains in bacterial anti-phage defense and expand our understanding of nucleotide signaling in antiviral immunity.

## Full Text Availability

The license terms selected by the author(s) for this preprint version do not permit archiving in PMC. The full text is available from the preprint server.

